# A comparison of clinical, lesion-based and connectome-based models of post-stroke depression: a prospective longitudinal study

**DOI:** 10.1016/j.nicl.2025.103911

**Published:** 2025-11-16

**Authors:** Nicolas Borderies, Suhrit Duttagupta, Thomas Tourdias, Sylvie Berthoz, Michel Thiebaut de Schotten, Igor Sibon

**Affiliations:** a Groupe d’Imagerie Neurofonctionnelle, Institut des Maladies Neurodégénératives 5293, Centre National de la Recherche Scientifique (CNRS), University of Bordeaux, Bordeaux 33076, France; b Univ. Bordeaux, INCIA CNRS, UMR 5287, Bordeaux F-33000, France; c Centre Hospitalier Universitaire (CHU) de Bordeaux, Neuroimagerie Diagnostique et Thérapeutique, Bordeaux 33076, France; d University Bordeaux, National Institute of Health and Medical Research (INSERM), Neurocentre Magendie, Bordeaux U1215, 33076, France; e Department of Psychiatry for Adolescents and Young Adults, Institut Mutualiste Montsouris, Paris F-75014, France; f Brain Connectivity and Behaviour Laboratory, Sorbonne Universities, Paris 75006, France; g Centre Hospitalier Universitaire (CHU) de Bordeaux, Unité Neurovasculaire, Bordeaux 33076, France

**Keywords:** Stroke, Depression, Connectome, Graph-theory, Predictive model, Network-symptom mapping

## Abstract

Post-stroke depression (PSD) is a frequent and disabling complication of ischemic stroke. Although clinical risk factors of PSD are well-established, the added predictive value of lesion and connectome-based brain-symptom mapping methods remains unclear. This study aimed to compare the predictive performance of clinical, lesion based, and network-based models of 3-months PSD in patients with minor ischemic stroke. We conducted a prospective longitudinal study including 263 patients with recent ischemic stroke. Clinical, radiological, and psychometric data were collected at baseline and at 3-month follow-up. Structural lesions were segmented on acute MRI and used to generate disconnectome maps and compute graph-theory metrics. Depressive symptoms severity at 3 months were measured using the CES-D scale. We developed six predictive models (clinical, radiological, gray-matter lesion, white matter disconnection, functional network disconnection, and topological graph features) using LASSO regression. Models performances were compared using the coefficient of determination (R^2^) and their combinations further examined through hierarchical regression. The clinical model demonstrated the best individual predictive performance (R^2^ = 23 %), identifying female sex, low cognitive status, poor functional outcome, and socioeconomic deprivation as significant predictors. Imaging-based models alone showed limited predictive power (R^2^ < 6 %), but identified significant associations for frontal and cerebellar lesions, somatomotor network disconnections, and altered network topology. A hierarchical model combining clinical, anatomical, and topological features significantly improved prediction (R^2^ = 31.6 %, p < 0.001), outperforming all individual models. In patients with minor ischemic stroke, PSD is most accurately predicted by clinical factors. However, combining lesion and network level neuroimaging features with clinical variables significantly enhances predictive accuracy. This multimodal approach supports the development of integrative, personalized models for PSD risk stratification and early intervention.

## Highlights

• Prospective cohort compares clinical, lesion, and connectome models for 3-month PSD.
• Frontal and cerebellar strokes associate with higher depressive symptoms.
• Network topology disruption (hub efficiency) contributes to PSD risk.
• Clinical variables outperform imaging; imaging adds modest, complementary info.
• Hierarchical integration of tiers improves prediction beyond single models.

## Graphical abstract

**Figure:**
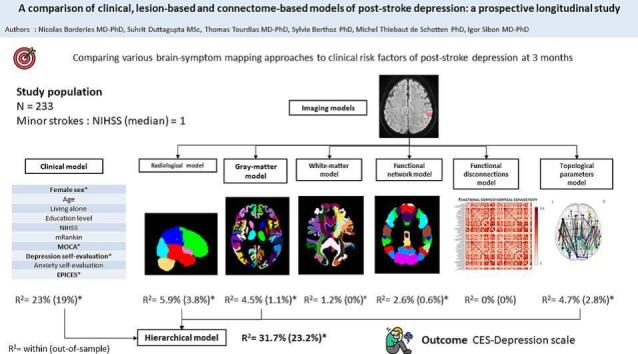

## 1 Introduction

Ischemic stroke is one of the leading causes of worldwide mortality and disability (Béjot et al., 2016, Feigin et al., 2021). Among the sequellae, post-stroke depression (PSD) is a very common complication affecting up to 27 % of patients (Liu et al., 2023a, Liu et al., 2023b) and is responsible for excess mortality and morbidity (Ayerbe et al., 2013, Kutlubaev and Hackett, 2014, Pan et al., 2008). A long-standing history of research on the pathophysiology of PSD draws a complex picture, viewing it as a product of multifaceted bio-psycho-social phenomena (Robinson and Jorge, 2016). Meta-analyses have highlighted five major risk factors of PSD: physical disability, cognitive impairment, history of prior depression, female sex, and lack of social support (Towfighi et al., 2017, Medeiros et al., 2020). Thus, medico-psycho-social factors seem to play an important role into the occurrence of PSD.

Conversely, regarding biological factors, stroke volume has been associated to PSD (Medeiros et al., 2020) but the role of stroke location, which should inform about the functional deficit, is still a matter of debate despite the development of advanced imaging sequences and methods of analysis (Wei et al., 2015). One possible explanation for the difficulty in identifying a specific brain region responsible for PSD is that it may stem from dysfunctions within interconnected brain networks rather than damage to a single critical region. This suggests that different stroke locations can disrupt these networks and contribute to the development of PSD. In line with current research on patients with major depressive disorder (MDD) demonstrating that major functional brain networks show altered connectivity patterns and abnormal topological organization (Cash et al., 2023, Chai et al., 2023, Gong et al., 2020, Gong and He, 2015), disruptions of these networks due to different stroke locations could contribute to the development of PSD.

To overcome the localisation limit of traditional lesion-symptom mapping, novel approaches have emerged to map symptoms to brain networks. Notably, an indirect method is to leverage the knowledge of the healthy human connectome and compute the pathological counterparts, the disconnectome – the set of structural or functional disconnections caused by a focal lesion delineated on routine MRI sequences (Fox, 2018). This approach succeeds in predicting long-term post-stroke clinical deficits in multiple domains better than the classical lesion-symptom approach (Thiebaut de Schotten et al., 2020). Recent high-powered studies using the disconnectome-mapping approach highlighted the role of frontal disconnections in PSD. However, the specific fibers or regions involved remain inconsistent across studies (Padmanabhan et al., 2019, Pan et al., 2022, Trapp et al., 2023, Weaver et al., 2023, Ríos et al., 2025). These discrepancies may stem from methodological factors, including population heterogeneity and spatial bias in distribution of lesions. Furthermore, like MDD, PSD is a complex syndrome that may involve the disruption of several neural networks, and no study has specifically evaluated the influence of neural network topological parameters. One alternative hypothesis is that stroke disconnections play only a minor role in the development of PSD compared to medico-psycho-social factors.

Importantly, while prior studies accounted for clinical confounding factors in their analysis pipeline, none of them compared the relative weight of clinical variables to imaging factors. Building predictive models comparing the contribution of each factor is a straightforward approach to inform the respective role of those factors. In this context, our objective was to compare different brain-symptom mapping approaches for PSD prediction (namely, gray-matter regions, white-matter tracts, functional networks and their disconnections, and topological parameters) and their additional value to clinical risk factors in the development of predictive models of 3-month PSD.

Thus, we developed 7 different predictive models, each one representing a specific type of information, with a graded level of complexity, to predict 3-months PSD. The clinical model (1) included most of the medico-psycho-social factors consistently reported in the literature. We built two localisionist models at different spatial resolution: the radiological model (2) incorporated the macroscopic stroke location for a coarse-grained approach, while the gray-matter model (3) included detailed gray-matter regions for a fine-grained approach. The disconnectome approach have been developed based on both structural and functional normative connectivity. Consequently, we developed a white-matter tract model (4) to assess structural disconnections and a functional disconnection model (5). However, these approaches assume that damage at the level of network links are relevant but it might be a more important disruption of the whole network that is relevant for PSD. We developed a model based major functional networks (6) described in the literature. Finally, we developed a model based on topological features (7) both at the global scale and local scale of functional networks, assuming PSD to reflect an emergent property of the brain networks. Predictive performance of each model was estimated and compared. In the end, we combined those models into a hierarchical one to assess the additive value of each source of information.

## 2 Materials and methods

### 2.1 Population

The population cohort was part of a larger prospective cohort recruited in Bordeaux (France) whose primary aim was to study the impact of a mobile mood-tracker app for the detection and prevention of post-stroke depression (MOTIVPOSDEP, ClinicalTrials: NCT04043052). Patients were recruited consecutively from September 2020 until September 2023. Participants included in the present analysis fulfilled the following inclusion criteria: (1) recent stroke (less or equal to 15 days ago) with clinical manifestations; (2) admitted to the stroke unit and discharged home; and (3) no prior severe handicap or prior cognitive impairment at inclusion (mRankin ≤ 3 and MOCA ≥ 16). The following exclusion criteria were also applied: (4) hemorrhagic stroke, transient ischemic attack, cerebral venous thrombosis, or any stroke mimic as a final diagnosis; (5) post-stroke severe cognitive impairment or severe aphasia (NIHSS item 9 ≥ 2); (6) pre-stroke severe mental disorder or psychoactive drug use during month preceding the stroke; (7) no diffusion-weighted MRI available at the acute stage; and (8) any handicap or environmental issue limiting the use of a mobile app.

### 2.2 Clinical, radiological and psychometric evaluation

Patients were evaluated at two time points: before acute ward discharge and 3 months later. Routine demographics and clinical measurements were collected at the acute stage, including age, sex, vascular risk factors, body mass index (BMI), pre-stroke IQ-code and modified Rankin Scale (mRS), reperfusion therapies, NIHSS, and MOCA scores. Additional data collected at the inclusion visit were radiological annotation of stroke location according to eight regions of interest: frontal, parietal, insular, temporal, and occipital lobes, basal ganglia, cerebellum and brainstem; a modified cerebral small vessel disease (cSVD) score without annotation for dilated perivascular spaces was computed (Staals et al., 2014); demographic data: education level, current or prior professional activity, social support, socio-economic status with the EPICES score (Labbe et al., 2015). Data on mood status were collected through three channels: (i) a clinical interview with a psychologist at inclusion and follow-up using the MINI (Sheehan, 1998); (ii) a visual analog scale between 0 and 10 for self-evaluation of anxiety and depression at inclusion; (iii) the completion of the CES-D scale (Center for Epidemiologic Studies – Depression) a validated self-report 20-item questionnaire to detect PSD symptoms (Meader et al., 2014) for the perceived level of depression at 3 months post-stroke,. Outcome scales were also evaluated at 3 months, including NIHSS, mRS, and MOCA. Antidepressant initiation at 3 months was also reported.

### 2.3 MRI acquisition procedure

Routine clinical MRI scans were collected during the acute stage as part of the standard diagnostic procedure. Images were acquired on a single SIEMENS Magnetron Area 1.5 Tesla scanner at the Bordeaux University Hospital. Acquisition parameters were as follows: the DWI sequence was parametrized with TR = 5000 ms, TE = 78 ms, flip angle = 90°, voxel size = 0.78 × 0.78x2.4 mm^3^, field of view = 270x245mm^2^; ADC maps were derived from b0 and b1000 images; the 2D-FLAIR sequence with TR = 9000 ms, TE = 120 ms, TI = 2500 ms, voxel size = 0.90 × 0.90 × 4.8 mm^3^, field of view = 230 × 230mm^2^; the T2 Gradient Echo sequence with TR = 948 ms, TE = 25 ms, flip angle = 15°, voxel size = 0.45 × 0.45 × 4.8 mm^3^, field of view = 180 × 230mm^2^.

### 2.4 MRI pre-processing pipeline

Ischemic lesions were delineated using a semi-automated procedure based on DWI b1000, ADC, and FLAIR maps. First, lesions were segmented based on the DWI images with the Acute-Stroke Detection Segmentation (ADS) toolbox (Liu et al., 2021). Second, all individual lesions were inspected and, if required, manually corrected by an experienced rater (N.B.) using ITK-SNAP 3.8 (www.itksnap.org). Spatial normalization was performed with the BCB-toolkit (https://storage.googleapis.com/bcblabweb/index.html). Normalized lesions were resampled to a voxel size of 2 × 2 × 2 mm^3^ using trilinear interpolation. Lesion volumes were derived from normalized images (to account for variability in brain volume). White matter hyperintensities (WMH) were automatically segmented on FLAIR native images with the Lesion Prediction Algorithm (LPA) available within the Lesion Segmentation Toolbox (LST) of SPM12 (Schmidt, 2017), and WMH volume was computed.

### 2.5 Statistical analyses

#### 2.5.1 Clinical and radiological statistics

All statistical analyses were performed on MATLAB software R2021b. Significance was set to P < 0.05. There was no imputation strategy for the missing data of patients lost to follow-up. Descriptive statistics were reported with median and interquartile range. The main outcome of this study was the CES-D total score and was taken as the dependent variable for all prediction models. Clinical and radiological models were constructed using a general linear model (GLM) with an intercept term and a beta coefficient for all independent variables considered as putative predictors. The clinical model included ten variables that have been associated with PSD or MDD in the literature (Medeiros et al., 2020): sex, age, living alone or not (a proxy for social support), education level (Backhouse et al., 2018), clinical severity (NIHSS score at inclusion), functional impairment (mRS score at inclusion), cognitive status (MOCA score at inclusion) (Butsing et al., 2024), depression and anxiety initial self-evaluation (Sagen et al., 2010), and socio-economic deprivation status (EPICES score, Labbe et al., 2015)(Guan et al., 2022). The radiological model included eight regions of interest: frontal, parietal, insular, temporal, and occipital lobes; basal ganglia, cerebellum and brainstem. Ordinary R^2^ was reported as a measure of goodness-of-fit. Model comparison was implemented with a comparison of Akaike Information Criterion (AIC) to account for model complexity. Out-of-sample predictions were computed by using a repeated k-fold cross-validation procedure (N_repetition_ = 10, N_fold_ = 10) with random partitions stratified according to CESD tertiles.

#### 2.5.2 Lesion and disconnection map generation

Lesion maps were generated by summing up all the binary lesion masks. The structural disconnectome (SDC) maps were generated using a deep-learning evaluation of the disconnectome through the BCB-toolkit (Matsulevits et al., 2023). The generated disconnectome map is probabilistic (between 0 and 100 %), reflecting the inter-individual variability. Disconnectome maps were thresholded ≥ 50 % to create binary masks and sampled at 2 × 2 × 2mm^3^.

#### 2.5.3 Brain-symptom mapping approach

Various brain-symptom mapping approaches were evaluated separately based on different atlas and network metrics described subsequently. The predictive performance of the models was evaluated individually with a LASSO regression, then hierarchical regression was performed on a combination of individual models to evaluate the effectiveness of a multimodal approach.

#### 2.5.4 MRI atlas-based analysis

Atlas-based parcellations were used for multivariate regressions to have a more coarse-grained level of analysis. Atlases were selected according to the following criteria: publicly available brain atlas in the MNI-152 space, probabilistic atlas constructed with a large sample of subjects, with an approximately similar number of parcels. The lesion load score for each region of interest (ROI) was the maximal probability of lesion when overlapping the lesion mask with the ROI mask. We used four atlases (detailed in Appendix A, Hansen et al., 2021, Rojkova et al., 2016) to quantify the impact of lesions onto gray-matter regions, white-matter tracts, functional networks and functional disconnections.

#### 2.5.5 Multivariate models: Lasso regression

Atlas-based multivariate models were all implemented with a LASSO-GLM using the glmnet package in MATLAB R2021b (see Supplementary Methods). Significance testing for each non-zero model was performed using an F-test versus a constant model. Coefficient of determination R^2^ was computed for within-sample and out-of-sample data (similarly as GLM models for clinical and radiological statistics). In order to compare the models, we chose the Akaike Information Criterion (AIC) as a simple metric that incorporates model accuracy and a model complexity term (related to the number of free parameters)(Akaike, 1974).

#### 2.5.6 Network-based models

Parcel-wise structural disconnectomes were computed through the use of the Lesion-Quantification toolkit (LQT) implemented in MATLAB R2021b (Griffis et al., 2021). A massive univariate approach was first used as implemented in the Network-Based Statistics (NBS) toolbox, then multivariate models were built similarly to the other atlas-based lasso regression models while using edges as regions of interest (see Supplementary Methods).

#### 2.5.7 Graph-theory measurements

The topological model included a list of graph-theory-based metrics computed with the Brain Connectivity Toolbox (BCT) in MATLAB R2021b. All measurements were computed for individual parcel-wise structural disconnectomes with the version adapted for weighted undirected graphs. We included parameters that summarize the major topological features of brain networks (see Supplementary Methods). The multivariate model was built similarly to atlas-based LASSO regression models with the 15 network metrics, with the exception that there was no constraint of non-negativity (since we could assume a positive or negative association with some network parameters).

#### 2.5.8 Mixture of models

Model combination was performed to assess whether mixing different multivariate models together could help improve the predictive performance of the best individual model. This strategy is relevant only if the different model predictions are dissimilar (i.e. errors of the different model predictions are not the same). We assessed model predictions' similarity with a Pearson correlation matrix. Then, we combined the model predictions using a hierarchical stepwise regression approach. Subordinate model predictions were included as independent predictor variables and the CES-D total score was the dependent variable. The null model included only an intercept term, and a forward algorithm added the best available subordinate model prediction only if this significantly improved the predictive performance of the superordinate model (according to the deviance criterion). The final model is obtained after convergence of the algorithm.

## 3 Results

### 3.1 Population characteristics

**Fig. 1:**
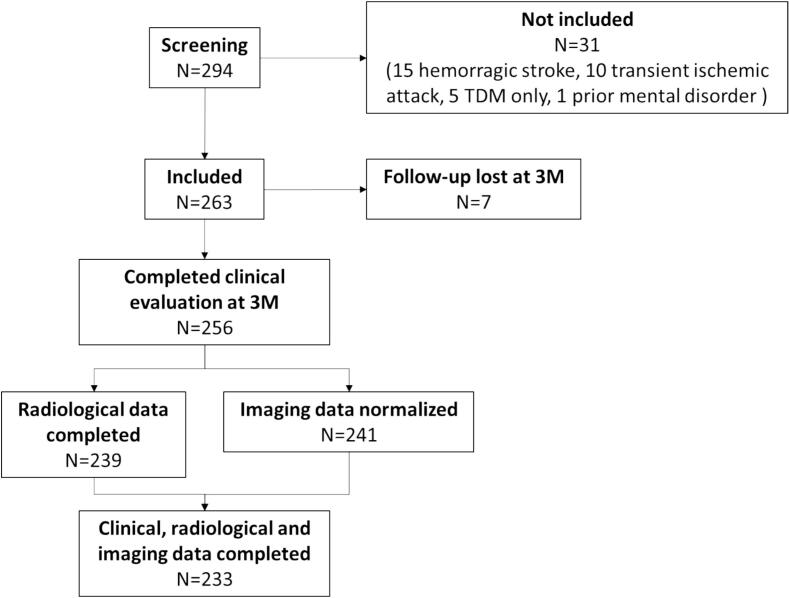
Flowchart of the study.

A total of 294 patients were screened according to the inclusion criteria and a total of 263 patients were identified (see Fig. 1). From this sample, the predictive models included the 233 patients with completed clinical, radiological and clinical data.

The study population included mainly men (70 %) of late-middle age (age = 58y ± 22y). Vascular risk factors followed the usual rate in stroke populations (see Table 1). At inclusion, clinical severity was low as measured by the initial (NIHSS median = 1 ± 3), as was functional impairment (mRS median = 0 ± 1). Stroke location was well-balanced between left and right hemispheres (50/54 %) and covered most of the arterial territories, including the vertebro-basilary territory, with the exception of the anterior cerebral artery (only two patients). These were small-volume strokes with a median volume of 1.08 mL (± 2.16), mostly monofocal (acute ischemic lesion number = 1 ± 1.25). Patients had minor post-stroke cognitive impairment (MOCA = 26 ± 3).

**Table 1:** Summary of main clinical, medical and stroke features of the population. Binomial variables are summarized by their number and proportion, continuous variables are summarized by their median and interquartile range. ACA = anterior cerebral artery, MCA = middle cerebral artery, PCA = posterior cerebral artery.

Variable	N/Median	Percent/IQR	Variable	N/Median	Percent/IQR
Demographic			Stroke features		
Sex (M/F)	164/69	70.39 %/29.61 %	Cortical strokes	110	47.21 %
Age	58	48–70	Subcortical strokes	96	41.20 %
Living Alone	47	20.17 %	Posterior fossa	54	23.18 %
Right-handed	210	90.13 %	ACA territory	2	0.86 %
Education Level	5	3–6	MCA territory	103	44.21 %
EPICES	13.61	7.1–22.63	PCA territory	58	24.89 %
IQCode	3	3–3.1	Cerebellar strokes	38	16.31 %
Medical			Brainstem strokes	18	7.73 %
Hypertension	117	50.21 %	Hemisphere (left/right)	117/126	50.21 % /54.08 %
Diabetes	25	10.73 %	Stroke volume (mL)	1.08	0.56–2.72
Hypothyroidism	14	6.01 %	Number of acute stroke lesion	1	1–2.25
Hypercholesterolemia	183	78.54 %	Cerebral Small Vessel Disease Score	0	0–1
Prior transient ischemic attack	14	6.01 %	White-Matter Hyperintensities volume (mL)	6.90	2.90–13.38
Prior stroke	16	6.87 %			
Coronary disease	13	5.58 %			
Cranial trauma	10	4.29 %			
Chronic illness	1	0.43 %			
Prior atrial fibrillation	15	6.44 %			
Smokers	78	33.48 %			
NIHSS (initial)	1	0–3			
NIHSS (discharge)	0	0–1			
mRankin (initial)	0	0–1			
mRankin (3-months)	1	0–1			
Intravenous thrombolysis	53	22.75 %			
Mechanical thrombectomy	8	3.43 %			
MOCA (discharge)	26	25–28			
MOCA (3-months)	26	25–28			
CES-D (3-months)	16	13–20.25			
Antidepressants (3-months)	11	4.72 %			

### 3.2 Clinical and radiological predictive models of depression

From the eight clinical factors tested based on their potential relevance for post-stroke depression in the literature, only four were significantly associated with the CES-D total score. Female sex was associated with greater depressive symptoms β = 1.38 (SE ± 0.70, t = 1.98, p = 0.05), as was poor socio-economic status (EPICES score β = 0.05, SE ± 0.02, t = 2.37, p = 0.02), lower cognitive ability (MOCA score β = −0.20, SE ± 0.12, t = −1.70, p = 0.09) and depression self-evaluation (β = 2.00, SE ± 0.37, t = 5.45, p = 1.32E-07). Other factors were not significantly associated with the CES-D score (see the Supplementary materials). The restricted GLM model with the four relevant predictive factors had a moderate goodness-of-fit with R^2^ = 23.03 % (more details in Supplementary Materials).

The radiological predictive model also comprised eight factors that were different anatomical stroke locations. We merged left and right locations because we found no significant effect of stroke laterality, putting apart bilateral lesions, on the CESD-D total score (β = -0.20, SE ± 0.70, t = -0.28, p = 0.77). Only two locations were significantly associated with the CES-D score: frontal strokes were associated with worse depressive symptoms (β = 2.84, SE ± 0.88, t = 3.23, p = 0.001), as were cerebellar strokes (β = 2.18, SE ± 0.95, t = 2.30, p = 0.02). The goodness of fit was weaker than the clinical model with R^2^ = 5.91 %.

There was no effect of ischemic lesion load on the CES-D score: neither acute ischemic lesion count (β = 0.02, SE ± 0.11, t = 0.21, p = 0.83), acute ischemic lesion volume (β = 0.003, SE ± 0.04, t = 0.08, p = 0.94), nor white-matter hyperintensities volume (β = 0.02, SE ± 0.03, t = 0.67, p = 0.51). A more integrative measurement of small-vessel disease radiological effects with the CSVD score wasn’t associated with the CES-D score (β = 0.03, SE ± 0.40, t = 0.07, p = 0.94). All regression coefficients Tables are available in the Supplementary Materials.

### 3.3 Lesion and disconnection maps

**Fig. 2:**
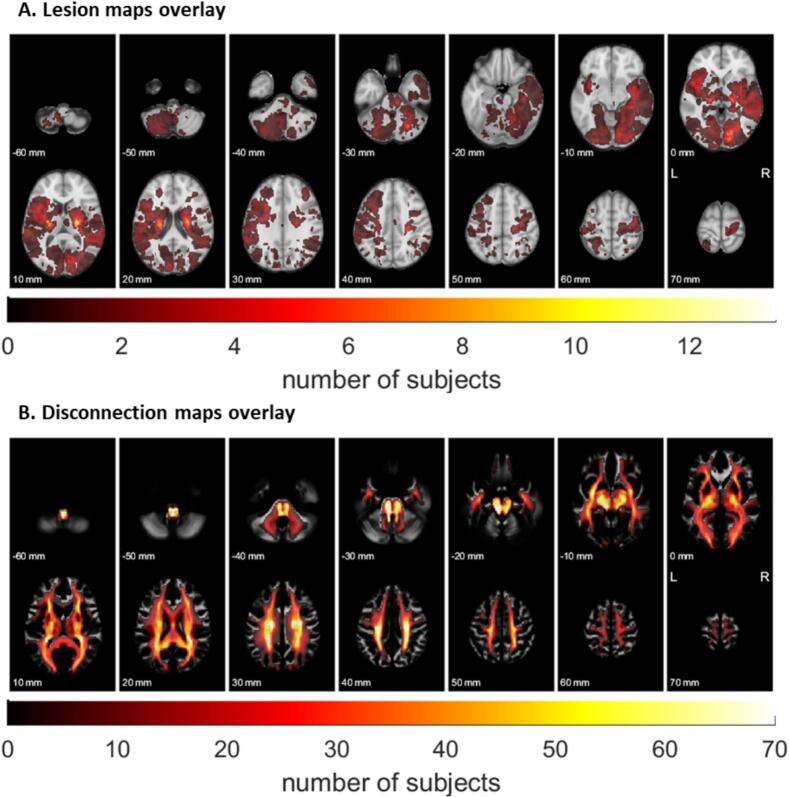
(A) Lesion maps overlay and (B) Disconnection maps overlay in the MNI-152 space.

Lesion frequency map is displayed in Fig. 2 which shows that the lesions were sparsely distributed across the brain with 30.7 % of voxels covered by at least one patient. In contrast, the disconnectome overlay map was more largely distributed across the white-matter with 81.9 % of voxels covered by at least one patient, with relative symmetry in the disconnections.

### 3.4 Lesion-based and dysconnectome-based multivariate predictive models of depression

Atlas-based multivariate LASSO regressions were performed to predict the CES-D scores. The gray-matter region-based regression model selected three variables after cross-validation: by order of importance, the left transverse temporal cortex, the right postcentral cortex, and the right precentral cortex. The predictive power of this model was low with in-sample R^2^ = 4.51 % (p = 0.01) and out-of-sample R^2^ = 1.10 %. The white-matter tract-based regression model selected only one variable after cross-validation: the left middle hand U-tract. The predictive power of this model was very low with in-sample R^2^ = 1.24 % (p = 0.09) and out-of-sample R^2^ = 0.04 %. The functional network region-based regression model selected two variables after cross-validation: the dorsal somato-motor network and the lateral somato-motor network. The predictive power of this model was low with in-sample R^2^ = 2.58 % (p = 0.05) and out-of-sample R^2^ = 0.58 %. Finally, the functionally disconnected region-based model didn’t keep any relevant variables in the regularized model after cross-validation, indicating zero predictive value.

### 3.5 Network-based predictive models of depression

**Fig. 3:**
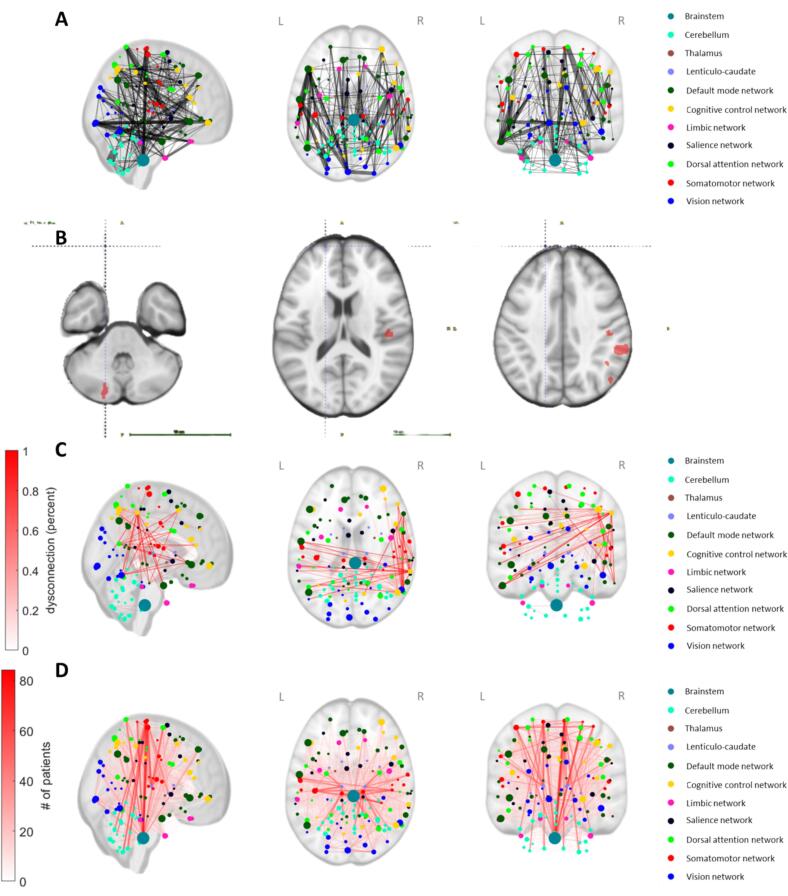
Parcel-wise connectome and disconnectome graphs. (A) normative connectome obtained from the LQT toolkit (Griffis et al., 2021), width of the edges corresponds to the number of fibers connecting two regions (the 1% less dense connections are not represented for the purpose of visibility), color represents network membership according to the Schaeffer atlas. (B) Example lesion segmentation (in red) of a single subject in the axial plane. (C) Corresponding example of disconnectome estimated from aforementioned subject lesion segmentation. (D) Group-level disconnectome overlay, width and color intensity represent the number of patients with a disconnection.

We computed the parcel-wise individual disconnectomes, with the disconnections overlay represented on Fig. 3, close to the normative connectome used in this study. The connection graph was homogeneously sampled with 96.64 % of edges lesioned in at least one patient. We ran Network-Based Statistics (NBS) to detect a potential subgraph of connected edges associated with the CES-D score, but no significant subgraph was found (Fmax = 5.76). Next, we used a multivariate LASSO regression model to predict depression scores based on all the edges of the parcel-wise disconnectomes (“dense” connectome model) or based on module-wise disconnections (within and between modules), but none of these models yielded any relevant variables after cross-validation. Finally, we computed 15 essential topological parameters of the parcel-wise disconnected graphs and performed a multivariate LASSO regression. This model selected four relevant parameters for depression prediction: a negative impact of maximal betweenness-centrality, default-mode network local efficiency, cognitive control network local efficiency, and salience network local efficiency (meaning lower topological parameters associated with higher depression scores). The predictive power of this model was similar to the gray-matter model with in-sample R^2^ = 4.74 % (p = 0.03) and out-of-sample R^2^ = 2.83 %.

### 3.6 Multivariate predictive models comparison and mixture

We performed a simple model comparison procedure between the six relevant multivariate LASSO regression models: the clinical, radiological, gray-matter region-based, white-matter tract-based, functional network region-based and network topology model. The clinical model was far better than other models based on imaging features with the best in-sample R^2^ and as well as the best AIC that takes model complexity into account (Table 2). Relative probabilities of the models showed that the clinical model had a strong support against other models (ΔAIC > 4 for all other models) and all models had at least moderate support against the null model (ΔAIC > 2), with the exception of the white-matter tract-based model.

**Table 2:** Model comparison of clinical, radiological, gray-matter region-based, white-matter tract-based, and network topology models. Second-to-last column depicts the relative probability of the model compared to the best model (computed according to AIC), and the last column depicts the relative probability of the model compared to the null (i.e. constant) model (computed according to AIC).

Model	R-squared (within)	R-squared (out)	p-value	AIC	P(model_i_)/P(best)	P(model_i_)/P(null)
Clinical	23.03 %	19.09 %	3.00E-12	722.83	1.00	3.20E + 11
Radiological	5.91 %	3.82 %	9.05E-04	765.62	5.12E-10	163.81
Gray-matter	4.51 %	1.10 %	0.01	771.07	3.35E-11	10.73
White-matter	1.24 %	0.04 %	0.09	774.91	4.91E-12	1.57
Functional networks	2.58 %	0.58 %	0.05	773.72	8.93E-12	2.86
Network topology	4.74 %	2.83 %	0.03	772.50	1.64E-11	5.26
Hierarchical model	31.65 %	23.20 %	1.33E-04	735.17	2.10E-4	6.71E + 08

There were no significant correlations between the network topological model and all other models. On the other hand, there was a weak correlation between the clinical model and other imaging models, and a moderate correlation between the four remaining imaging models, but with all the correlation coefficients ρ being under 0.7 indicated that the collinearity would not greatly prevent fitting a hierarchical model. The correlation matrix is available in the Supplementary Materials. Therefore, we performed a hierarchical stepwise regression to predict the CES-D score based on a combination of subordinate models. The stepwise hierarchical model only selected four models: the clinical, radiological, the gray-matter region-based, and the network topological model (detailed weights for each subordinate model in Supplementary Table 6). The predictive performance of the hierarchical model was better than the best single model with in-sample R^2^ = 31.65 % (F-test comparing the hierarchical model to the best single model: F = 1.64, p = 6.123e-02) and out-of-sample R^2^ = 23.20 %, and a strong support with ΔAIC > 4 against all single models (AIC = 735.2), except the clinical model (AIC = 722.8) (Table 2).

## 4 Discussion

In the current study, we evaluated the performance of several recent brain-symptom mapping methods based on MRI atlas (gray-matter regions, white-matter tracts, functional networks and disconnections, and topological parameters) to predict PSD. Most of these methods yielded significant associations (except the functional disconnection model) but always with a low predictive value (R^2^ < 10 %). None of the brain imaging models were better than the clinical prediction model. However, combining these brain imaging models with the clinical model significantly improved the prediction of PSD compared to clinical variables alone (R^2^ = 31.65 % vs. 23.03 %). In particular, the radiological, the gray-matter region-based, and the network topological model provided different types of information on PSD risk that seem to be complementary. To our knowledge, the present study is the first to demonstrate the superiority of such a combined approach for the prediction of PSD.

### 4.1 Clinical model of PSD

We first built a clinical model predicting PSD, identifying expected risk factors such as female sex, cognitive impairment (MOCA), socio-economic deprivation (EPICES) and initial depression self-evaluation. Lower cognitive ability is an established risk factors (Hackett and Anderson, 2005, Kutlubaev and Hackett, 2014, Towfighi et al., 2017), while socio-economic influences are more debated: although education level is widely associated with PSD, the independent role of poor economic status remains inconsistent across studies (Backhouse et al., 2018), likely due to cultural and systemic differences. Functional impairment has been consistently associated with PSD in other studies (Towfighi et al., 2017), we only found a non-significant trend for the association with modified Rankin score and no effect of NIHSS score but it might relate to a floor effect in our minor stroke population. Initial depressive symptomatology measured with a self-evaluation scale was the most important clinical factor, this reinforces the idea that psychological states or traits, prior or during the acute stage of stroke, play a major role in the development of chronic depressive symptomatology. Patients more depressed initially may be those with less effective coping strategy in their daily life after acquisition of a neurological deficit. Notably, clinical and socio-demographic variables had a stronger impact on PSD than any imaging variable, even in our carefully selected cohort, emphasizing their major role in risk assessment, especially after minor strokes.

### 4.2 Imaging models of PSD

Our crude radiological model highlighted frontal and cerebellar lesions as associated with PSD. Frontal involvement is well-established (Nickel and Thomalla, 2017), while the cerebellum's role has been underrecognized, partly due to lesion distribution biases. Only one voxel-based study restricted to cerebellar strokes showed a left posterior cerebellar association (Kim et al., 2017). Cerebellar strokes can disrupt widespread cortical networks via functional diaschisis (Duttagupta et al., 2024). Our finding aligns with the cerebellar cognitive-affective syndrome (CCAS, Schmahmann and Sherman, 1998).

Atlas-based multivariate models consistently implicated the somatomotor network: within the functional network model, the right pre/postcentral gyri within the gray-matter model, and somatomotor U-tracts within the white-matter model. This aligns with evidence linking somatomotor network dysfunction to MDD (Javaheripour et al., 2021). This damage may likely worsen depression indirectly through subtle motor or sensory deficits. Therefore, promising non-invasive neuromodulation techniques targeting the motor circuits which are currently developed for motor recovery (Keser et al., 2023) might have collateral positive effect on mood. More generally, individualized targeting of brain networks for PSD based on lesion/disconnections might be an appealing approach as it is similarly developed for major depression (Cash et al., 2021). We also found PSD associations with the left transverse temporal gyrus, possibly via impaired sound processing. Our models did not capture cerebellar strokes, possibly due to limited cerebellar parcellation in the atlas used. The functional disconnection model showed no significant results, possibly because parcel-wise normative connectomes are too crude (Thiebaut de Schotten et al., 2020). While direct resting-state fMRI could improve prediction (Balaev et al., 2018, Jaywant et al., 2022, Zhang et al., 2018), its clinical feasibility is limited.

Finally, several structural connectome topological parameters were linked to PSD. Globally, only maximal betweenness-centrality (reflecting disruption of network hubs) was associated with PSD, consistent with findings in MDD (Gong and He, 2015) and post-stroke cognitive impairment (Aben et al., 2019). Locally, decreased efficiency in the default mode, cognitive control, and salience networks predicted PSD. This echoes fMRI studies in MDD (Chai et al., 2023) and converging studies pointing towards a depression network (Padmanabhan et al., 2019), largely overlapping with the default mode et cognitive control networks. These effects were independent of edge density, highlighting the specific role of topological disorganization, irrespective of the spatial damage. Graph theory can help to understand robustness of a network to damages and it might provide critical information about the synaptic plasticity that occurs after stroke, as this is a potential driver of PSD remission (Fang et al., 2025). This could inform in the future neuromodulation techniques such as vagus nerve stimulation, proven to be effective in major depressive disorder and known to have large-scale plasticity effects (Kamel et al., 2022).

### 4.3 Models comparison and combination

Comparison of the imaging models led to a similar level of predictive value (from 1 % to 6 % of R^2^) with correlated individual predictions, suggesting that the anatomical information extracted from these models is close. One exception was the topological model that had uncorrelated predictions, which relates to the fact that it is based on topological features. However, predictions from the clinical model and the imaging models were dissociated, which enabled us to construct a hierarchical *meta*-model that combined clinical, radiological, lesion, and topological features; this hierarchical model improved the predictive performance of the clinical model and was significantly better than any single model. Therefore, a combination of various features can help improve the prediction of PSD. This is consistent with the multifactorial origin of PSD and the necessity to incorporate different sources of information in order to make accurate predictions. Interestingly, two recent studies suggested that combining imaging and clinical data outperforms traditional methods in order to predict post-stroke motor outcome (Olafson et al., 2024) and also functional outcome (Liu et al., 2023a, Liu et al., 2023b). Another complementary approach would be to integrate the neurotransmitter system dysregulated by a stroke as suggested by a recent study (Alves et al., 2025) to predict PSD and even help to select the appropriate antidepressant medication. Such an approach paves the way to personalized prediction models of PSD in order to select high-risk patients for psychotherapeutic and/or pharmacological preventive intervention.,

### 4.4 Limitations

The present work comprises several limitations. First, our stroke cohort is not fully representative of the global stroke population since patients were young, had minor strokes, had little disability, and no history of current or recent (over the past 6 months) mood or anxiety disorders or associated medication. Additionally, there were few strokes in the anterior cerebral artery territory and left temporal lobe. Consequently, generalization towards the overall stroke population is limited, although it is common practice to selectively choose stroke candidates in PSD studies in order to disambiguate the various intricate risk factors (Towfighi et al., 2017). Additionally, small stroke lesions minimize the natural spatial autocorrelation of stroke, which is problematic for spatial inference. Second, we used an indirect measurement of structural and functional disconnectomes. A direct measurement of the structural connectome with DTI imaging might provide more accurate information on the damaged fibers at the individual level. However, previous validation studies demonstrated a relatively good agreement between indirect structural disconnectomes and direct measurement with DTI (Foulon et al., 2018), and the point of this approach was precisely to explore the predictive power of routine MRI procedure. Third, we computed indirect disconnections based on the acute ischemic stroke lesion, but this approach neglects other sources of brain damage that might contribute to the risk of PSD. Importantly, cerebral small vessel disease (cSVD) lesions have been associated with PSD in some studies (Castello et al., 2022, Zhang et al., 2017) as well as with “vascular depression”, which denotes late-onset depression in people with vascular risk factors, potentially because of fronto-limbic white-matter disconnections (Taylor et al., 2013). We did not find any effect of simple cSVD imaging markers (cSVD score, white-matter hyperintensity volume) in our study, but our population of young subjects had a low amount of cSVD lesions, and the effect might be more driven by strategic disconnections. Fourth, we used an atlas-based approach to build multivariate models, which limits the dimensionality of the predictor variables but raises the issue of the parcellation scheme choice. There are plenty of atlases available for MRI neuroimaging that use various parcellations based on different features and different borders for the same regions, which is known as the atlas-concordance problem (Revell et al., 2022). We believe that this should not drastically impact our results because we used atlases in agreement fuelled with probabilistic maps built on large population samples. The advantage of the atlas-based approach is to limit the dimensionality of the dataset, thereby limiting potential spurious associations. Additionally, it is a mandatory step in graph-based analysis of the connectome, so it was a better comparator to lesion-based and disconnectome-based models.

## 5 Conclusion

In conclusion, we compared several predictive models of post-stroke depression (PSD) in a population of minor ischemic strokes. The clinical model was the best single model, but macroscopic radiological location was also informative, revealing a higher risk for frontal and cerebellar strokes, and more detailed gray-matter regions and white-matter tracts brought additional predictive value. Beyond simple stroke location, alteration of structural network topology with critical disconnection of network hubs was also predictive of PSD. The combination of these models significantly improved predictive performance beyond all subordinate models, supporting the use of multimodal predictive models for PSD. Future studies are necessary to assess the external validity of such models and evaluate their clinical impact in terms of better screening and prevention of PSD.

## CRediT authorship contribution statement

**Nicolas Borderies:** Writing – review & editing, Writing – original draft, Visualization, Software, Methodology, Formal analysis, Data curation. **Suhrit Duttagupta:** Writing – review & editing, Formal analysis. **Thomas Tourdias:** Writing – review & editing, Supervision, Software, Resources, Project administration, Methodology, Investigation, Funding acquisition, Conceptualization. **Sylvie Berthoz:** Writing – review & editing, Validation, Supervision, Project administration, Methodology, Investigation, Funding acquisition, Conceptualization. **Michel Thiebaut de Schotten:** Writing – review & editing, Supervision, Software, Methodology, Conceptualization. **Igor Sibon:** Writing – review & editing, Validation, Supervision, Resources, Project administration, Investigation, Funding acquisition, Data curation, Conceptualization.

## Declaration of competing interest

The authors declare that they have no known competing financial interests or personal relationships that could have appeared to influence the work reported in this paper.

## Data Availability

Data will be made available on request.
