# Supplementary material for: A comparison of clinical, lesion-based and connectome-based models of post-stroke depression: a prospective longitudinal study
Source: Neuroimage Clin. 2025 Nov 16;48:103911. doi: 10.1016/j.nicl.2025.103911 (PMC12671036; doi:10.1016/j.nicl.2025.103911)
Supplement: Supplementary Data 1 [file mmc1.docx]

# Supplementary materials

## Supplementary methods

### MRI Atlas-based analysis

The gray-matter regions atlas was the IIT-GM-Desikan atlas which is a probabilistic version of the Desikan atlas with 84 labels depicting cortical and subcortical gray matter regions. The white-matter tracts atlas was a combination of the BCBToolkit atlas that contains 68 tracts that satisfactorily cover associative tracts, with a particular focus on frontal tracts (Foulon et al., 2018; Rojkova et al., 2016), augmented with the PANDORA white-matter atlas (the version based on the TractSeg algorithm) that satisfactorily cover projection tracts— cortico-subcortical bundles in particular—and was built with a large population of subjects (N=2443), including non-redundant tracts (Hansen et al., 2021); this led to a combined white-matter atlas of 96 tracts. The functional network atlas was the Dworetsky atlas, a probabilistic atlas based on a consensus identification of core regions forming 14 resting-state networks. The lesion score for each region-of-interest was the maximal lesion probability crossed by the individual lesion mask.

The functional disconnection maps were created in two steps: first, lesion maps were projected onto the Desikan-Killiany atlas with computation of the lesion proportion for each region-of-interest; secondly, a normative parcel-wise functional connectome extracted from the HCP thanks to the ENIGMA toolbox (with only positive coefficients stored) was used to compute the regions functionally disconnected from the lesioned parcels. Mathematically, the disconnection score for each parcel was computed as follows:

$disconnection \left( {parcel}_{i} \right)=\frac{1}{n\left( parcels \right)}\times\sum_{j=all parcels\neq i} lesion proportion\left( {parcel}_{j} \right)\times correlation\left( {parcel}_{i},{parcel}_{j} \right)$

### Multivariate models: lasso regression

This approach simultaneously performs a regularized regression, i.e. provide a shrinked estimator of beta coefficients that prevent overfitting, and a feature selection, i.e. keep only the minimal number of non-zero coefficients to explain the data; this is particularly well-suited for prediction of data based on sparse predictors (large number of predictors compared to data lines, the majority of data points equals to zero), such as atlas-based probabilistic lesions. Mathematically, this corresponds to minimizing the least-square residuals with an additional penalty term, the sum of absolute value of beta coefficients (i.e. the L1-norm) weighted by the hyperparameter λ, realizing a trade-off between accuracy and complexity of the model. λ tuning was realized with a repeated 10-fold cross-validation procedure; the optimal λ value was the one that minimized the mean squared error on the out-of-sample data. Importantly, an additional constraint of non-negativity was set for beta coefficients such that variables representing damaged brain structures could only explain an increase in the CES-D depression score.

### Network-based models

Essentially, the Lesion-Quantification toolkit (LQT) enables to compute an indirect disconnection matrix with nodes representing gray-matter parcels and edges representing fibers connecting two parcels. The default parcellation scheme is based on the Schaeffer 100 cortical regions atlas combined with the AAL atlas for subcortical regions and cerebellum and with the brainstem mask from the Harvard-Oxford Subcortical Atlas, thus the whole brain is divided in 135 gray-matter regions. Then, the structural connection strengths are estimated through the use of a normative tractography atlas (HCP-842 atlas in MNI space), the lesion masks are projected on this atlas, and we count the number of streamlines connecting terminating into two parcels that are damaged. The normative structural connectivity matrix contains 1533 edges which represent 17% of the 9035 possible connections.

A massive univariate approach was first used as implemented in the Network-Based Statistics (NBS) toolbox. This methodology performs a univariate statistical test on every edge of the network with a multiple-comparison correction procedure that takes into account the topological distance between connected edges. We constructed a GLM regression with an intercept and a beta coefficient for the CES-D score for all edges, and performed a F-test on the beta coefficient for significance. The minimal threshold statistic at the edge-level was set to 2.5, and family-wise error rate p(FWER) was set to 0.05 at the subgraph level, computed through 5000 random permutations. We tested both hypotheses of extensive effect and intensive effect at the subgraph level.

The multivariate models were built similarly to the other atlas-based lasso regression models. A first “dense” connectome model was estimated with all the 1533 edges as dependent variables. Because this model was largely oversized in terms of number of predictors, a second “modular” connectome model was estimated by averaging the connections within modules and between modules leading to only 81 edges. A total of 11 modules were considered for averaging across the edges: the seven functional networks defined by the Yeo atlas that embed the 100 Schaeffer cortical parcellation, and four subcortical structures (lenticulo-caudate nucleus, thalamus, cerebellum and brainstem).

### Graph-theory measurements

A set of 15 network metrics was selected to summarize the major topological features of brain networks. Thus, we computed two scale parameters: the mean degree and the density of the graph (to assess whether the extent of disconnection at the graph-level explain the clinical outcome); three clustering parameters: the mean clustering coefficient, the modularity and the transitivity of the graph (to assess whether the alteration of modular organization within the connectome explain the clinical outcome); two parameters measuring the core/periphery structure : the assortativity coefficient and the area-under-the-curve of the rich-club coefficient plot (to assess whether alteration of the core “rich-club” architecture explain the clinical outcome); two parameters measuring the importance of central nodes: the maximal degree and the maximal betweeness-centrality (to assess whether lesions that disconnected central hubs explain the clinical outcome); one parameter of global efficiency of the graph: the characteristic path length (to assess whether alteration of the global efficiency explain the clinical outcome); and five local efficiency parameters within the networks associated with depression in the literature : default-mode, cognitive control, limbic, salience, and dorsal attention network local efficiency (to assess whether alteration of local efficiency within a module explain the clinical outcome). Detailed definitions of the network metrics are provided in the supplementary materials.

## Supplementary results

*Supplementary Table 1. Gray Matter region atlas used in the atlas-based analysis. Here we report the region's volume, the number of subjects with at least >1% probability of lesion, and the average lesion probability within this population of subjects.*

| ***Gray-matter Region*** | ***Volume (mL)*** | ***Number of subjects*** | ***Average lesion probability*** |
| --- | --- | --- | --- |
| Left_Accumbens_area | 0,789 | 1 | 0,014705882 |
| Left_Amygdala | 3,623 | 0 |  |
| Left_Caudate | 5,818 | 18 | 1 |
| Left_Cerebellum_Cortex | 92,215 | 25 | 0,881043812 |
| Left_Hippocampus | 7,348 | 3 | 0,682539683 |
| Left_Pallidum | 1,394 | 14 | 0,790703199 |
| Left_Putamen | 6,22 | 19 | 0,974978118 |
| Left_Thalamus_Proper | 8,36 | 36 | 1 |
| Right_Accumbens_area | 0,953 | 0 |  |
| Right_Amygdala | 3,896 | 7 | 0,64468416 |
| Right_Caudate | 5,499 | 14 | 1 |
| Right_Cerebellum_Cortex | 90,662 | 40 | 0,904871099 |
| Right_Hippocampus | 6,948 | 11 | 0,912655972 |
| Right_Pallidum | 1,272 | 15 | 0,768487614 |
| Right_Putamen | 6,464 | 17 | 0,942611191 |
| Right_Thalamus_Proper | 9,152 | 40 | 0,99875 |
| ctx_lh_bankssts | 9,693 | 9 | 0,626707658 |
| ctx_lh_caudalanteriorcingulate | 6,538 | 0 |  |
| ctx_lh_caudalmiddlefrontal | 14,42 | 15 | 0,806795508 |
| ctx_lh_cuneus | 9,865 | 22 | 0,38135859 |
| ctx_lh_entorhinal | 7,203 | 0 |  |
| ctx_lh_frontalpole | 2,562 | 0 |  |
| ctx_lh_fusiform | 31,348 | 14 | 0,553518107 |
| ctx_lh_inferiorparietal | 33,581 | 27 | 0,561191044 |
| ctx_lh_inferiortemporal | 26,959 | 13 | 0,24442327 |
| ctx_lh_insula | 16,976 | 17 | 0,798902429 |
| ctx_lh_isthmuscingulate | 6,036 | 6 | 0,299130588 |
| ctx_lh_lateraloccipital | 21,356 | 29 | 0,553950919 |
| ctx_lh_lateralorbitofrontal | 16,453 | 2 | 1 |
| ctx_lh_lingual | 18,952 | 25 | 0,424479815 |
| ctx_lh_medialorbitofrontal | 12,821 | 1 | 1 |
| ctx_lh_middletemporal | 29,829 | 11 | 0,36229449 |
| ctx_lh_paracentral | 7,877 | 3 | 0,477929523 |
| ctx_lh_parahippocampal | 5,77 | 3 | 0,76000001 |
| ctx_lh_parsopercularis | 11,123 | 11 | 0,645937256 |
| ctx_lh_parsorbitalis | 5,861 | 2 | 0,232512314 |
| ctx_lh_parstriangularis | 9,823 | 7 | 0,586849081 |
| ctx_lh_pericalcarine | 6,496 | 15 | 0,650664117 |
| ctx_lh_postcentral | 19,321 | 29 | 0,703641288 |
| ctx_lh_posteriorcingulate | 9,737 | 4 | 0,512063105 |
| ctx_lh_precentral | 25,626 | 30 | 0,79675655 |
| ctx_lh_precuneus | 22,556 | 21 | 0,508146532 |
| ctx_lh_rostralanteriorcingulate | 8,539 | 0 |  |
| ctx_lh_rostralmiddlefrontal | 31,508 | 9 | 0,615371717 |
| ctx_lh_superiorfrontal | 44,262 | 9 | 0,225132758 |
| ctx_lh_superiorparietal | 27,576 | 28 | 0,620511057 |
| ctx_lh_superiortemporal | 27,343 | 15 | 0,800823011 |
| ctx_lh_supramarginal | 25,529 | 19 | 0,535812033 |
| ctx_lh_temporalpole | 6,898 | 0 |  |
| ctx_lh_transversetemporal | 3,878 | 12 | 0,678846365 |
| ctx_rh_bankssts | 9,319 | 11 | 0,604891634 |
| ctx_rh_caudalanteriorcingulate | 6,548 | 0 |  |
| ctx_rh_caudalmiddlefrontal | 13,221 | 13 | 0,614267935 |
| ctx_rh_cuneus | 9,439 | 19 | 0,543539724 |
| ctx_rh_entorhinal | 6,041 | 3 | 0,378648234 |
| ctx_rh_frontalpole | 3,076 | 0 |  |
| ctx_rh_fusiform | 30,102 | 27 | 0,599314537 |
| ctx_rh_inferiorparietal | 36,215 | 29 | 0,507963855 |
| ctx_rh_inferiortemporal | 30,833 | 24 | 0,305501473 |
| ctx_rh_insula | 15,571 | 21 | 0,75843791 |
| ctx_rh_isthmuscingulate | 5,794 | 8 | 0,619905832 |
| ctx_rh_lateraloccipital | 18,493 | 27 | 0,493367672 |
| ctx_rh_lateralorbitofrontal | 15,878 | 2 | 0,039473685 |
| ctx_rh_lingual | 18,368 | 25 | 0,590729075 |
| ctx_rh_medialorbitofrontal | 12,059 | 0 |  |
| ctx_rh_middletemporal | 35,09 | 16 | 0,478071984 |
| ctx_rh_paracentral | 8,723 | 5 | 0,773744707 |
| ctx_rh_parahippocampal | 5,199 | 9 | 0,703323086 |
| ctx_rh_parsopercularis | 9,497 | 12 | 0,424547471 |
| ctx_rh_parsorbitalis | 6,583 | 1 | 0,024390243 |
| ctx_rh_parstriangularis | 9,481 | 3 | 0,169731504 |
| ctx_rh_pericalcarine | 7,049 | 22 | 0,492041509 |
| ctx_rh_postcentral | 18,643 | 28 | 0,741586663 |
| ctx_rh_posteriorcingulate | 8,794 | 3 | 0,869090915 |
| ctx_rh_precentral | 23,676 | 28 | 0,818881296 |
| ctx_rh_precuneus | 24,031 | 16 | 0,476483979 |
| ctx_rh_rostralanteriorcingulate | 8,068 | 0 |  |
| ctx_rh_rostralmiddlefrontal | 31,915 | 5 | 0,716176471 |
| ctx_rh_superiorfrontal | 44,287 | 9 | 0,299304555 |
| ctx_rh_superiorparietal | 26,741 | 35 | 0,532016814 |
| ctx_rh_superiortemporal | 28,038 | 16 | 0,698744118 |
| ctx_rh_supramarginal | 25,891 | 28 | 0,603831948 |
| ctx_rh_temporalpole | 6,875 | 3 | 0,618200836 |
| ctx_rh_transversetemporal | 2,93 | 12 | 0,612659322 |

*Supplementary Table 2. White-Matter tract atlas used in the atlas-based analysis. Here we report the tract volume, the number of subjects with at least >1% probability of lesion, and the average lesion probability within this population of subjects.*

| **Tract** | **Volume (mL)** | **Number of subjects** | **Average lesion probability** |
| --- | --- | --- | --- |
| Anterior_Commissure | 66,483 | 77 | 0,413432477 |
| Anterior_Thalamic_Projections_Left | 170,167 | 72 | 0,860898641 |
| Anterior_Thalamic_Projections_Right | 178,271 | 79 | 0,745316457 |
| Arcuate_Anterior_Segment_Left | 58,015 | 32 | 0,681250003 |
| Arcuate_Anterior_Segment_Right | 104,41 | 50 | 0,655199999 |
| Arcuate_Long_Segment_Left | 96,311 | 38 | 0,662105263 |
| Arcuate_Long_Segment_Right | 66,221 | 47 | 0,617838939 |
| Arcuate_Posterior_Segment_Left | 66,532 | 14 | 0,764285717 |
| Arcuate_Posterior_Segment_Right | 81,602 | 27 | 0,684438539 |
| Cingulum_Left | 165,209 | 58 | 0,501724138 |
| Cingulum_Right | 109,785 | 21 | 0,61809524 |
| Corpus_callosum | 1201,132 | 203 | 0,790456439 |
| Cortico_Spinal_Left | 83,843 | 82 | 0,767024392 |
| Cortico_Spinal_Right | 104,592 | 103 | 0,787883496 |
| Face_U_tract_Left | 11,11 | 14 | 0,486880475 |
| Face_U_tract_Right | 10,114 | 10 | 0,59069767 |
| Fornix | 64,181 | 42 | 0,511619049 |
| Frontal_Aslant_Tract_Left | 88,133 | 35 | 0,634285714 |
| Frontal_Aslant_tract_Right | 87,674 | 38 | 0,607894734 |
| Frontal_Commissural | 501,011 | 83 | 0,728915661 |
| Frontal_Inferior_longitudinal_Left | 36,399 | 16 | 0,691326533 |
| Frontal_Inferior_longitudinal_Right | 51,289 | 21 | 0,543809524 |
| Frontal_Orbito_Polar_Left | 24,021 | 3 | 0,482993215 |
| Frontal_Orbito_Polar_Right | 26,027 | 0 |  |
| Frontal_Superior_Longitudinal_Left | 70,446 | 23 | 0,753913042 |
| Frontal_Superior_Longitudinal_Right | 66,831 | 25 | 0,732800003 |
| Fronto_Insular_tract1_Left | 1,098 | 5 | 0,155999999 |
| Fronto_Insular_tract1_Right | 2,225 | 3 | 0,320000003 |
| Fronto_Insular_tract2_Left | 1,963 | 6 | 0,333333333 |
| Fronto_Insular_tract2_Right | 10,49 | 5 | 0,6 |
| Fronto_Insular_tract3_Left | 11,003 | 13 | 0,526153854 |
| Fronto_Insular_tract3_Right | 19,864 | 18 | 0,681111107 |
| Fronto_Insular_tract4_Left | 14,156 | 20 | 0,715000006 |
| Fronto_Insular_tract4_Right | 16,577 | 26 | 0,741538462 |
| Fronto_Insular_tract5_Left | 12,025 | 21 | 0,692380957 |
| Fronto_Insular_tract5_Right | 18,629 | 32 | 0,820091566 |
| Fronto_Marginal_tract_left | 20,763 | 0 |  |
| Fronto_Marginal_tract_right | 30,279 | 0 |  |
| Fronto_pontine_tract_left | 116,444 | 91 | 0,749614073 |
| Fronto_pontine_tract_right | 115,885 | 94 | 0,714925737 |
| Handinf_U_tract_Left | 24,662 | 25 | 0,697142862 |
| Handinf_U_tract_Right | 20,85 | 28 | 0,557142856 |
| Handmid_U_tract_Left | 2,877 | 15 | 0,320833328 |
| Handmid_U_tract_Right | 10,216 | 23 | 0,637372805 |
| Handsup_U_tract_Left | 27,354 | 24 | 0,749999997 |
| Handsup_U_tract_Right | 20,687 | 26 | 0,722135011 |
| Inferior_Fronto_Occipital_fasciculus_Left | 152,271 | 47 | 0,826158225 |
| Inferior_Fronto_Occipital_fasciculus_Right | 147,489 | 45 | 0,802222227 |
| Inferior_Longitudinal_Left | 178,634 | 40 | 0,741200002 |
| Inferior_Longitudinal_Right | 174,805 | 45 | 0,837169372 |
| Inferior_cerebellar_peduncle_left | 40,262 | 21 | 0,70915053 |
| Inferior_cerebellar_peduncle_right | 35,448 | 36 | 0,715717451 |
| Middle_cerebellar_peduncle | 107,563 | 58 | 0,797711574 |
| Optic_Radiations_Left | 55,316 | 41 | 0,754146342 |
| Optic_Radiations_Right | 42,182 | 46 | 0,694956519 |
| Paracentral_U_tract_Left | 5,087 | 0 |  |
| Paracentral_U_tract_Right | 3,416 | 3 | 0,453333348 |
| Parieto_occipital_pontine_left | 139,476 | 109 | 0,835523081 |
| Parieto_occipital_pontine_right | 137 | 124 | 0,8356322 |
| Striato_fronto_orbital_left | 32,757 | 13 | 0,708560659 |
| Striato_fronto_orbital_right | 26,764 | 7 | 0,290757023 |
| Striato_occipital_left | 89,206 | 67 | 0,643623463 |
| Striato_occipital_right | 88,673 | 73 | 0,662059128 |
| Striato_parietal_left | 202,191 | 98 | 0,824185411 |
| Striato_parietal_right | 206,443 | 101 | 0,900734807 |
| Striato_postcentral_left | 79,303 | 78 | 0,786216696 |
| Striato_postcentral_right | 74,847 | 85 | 0,85075775 |
| Striato_precentral_left | 104,761 | 83 | 0,783518849 |
| Striato_precentral_right | 97,707 | 86 | 0,873948489 |
| Striato_prefrontal_left | 165,057 | 59 | 0,669322784 |
| Striato_prefrontal_right | 164,502 | 55 | 0,646091339 |
| Striato_premotor_left | 60,863 | 43 | 0,674444636 |
| Striato_premotor_right | 52,015 | 44 | 0,627565666 |
| Superior_Londgitudinal_Fasciculus_III_Left | 128,434 | 57 | 0,698808942 |
| Superior_Londgitudinal_Fasciculus_III_Right | 197,161 | 63 | 0,772297639 |
| Superior_Londgitudinal_Fasciculus_II_Left | 184,107 | 59 | 0,78359322 |
| Superior_Londgitudinal_Fasciculus_II_Right | 191,621 | 59 | 0,810983051 |
| Superior_Londgitudinal_Fasciculus_I_Left | 171,462 | 38 | 0,696210527 |
| Superior_Londgitudinal_Fasciculus_I_Right | 156,993 | 43 | 0,650604653 |
| Superior_cerebellar_peduncle_left | 48,12 | 63 | 0,747438733 |
| Superior_cerebellar_peduncle_right | 47,814 | 84 | 0,705545639 |
| Thalamo_occipital_left | 76,255 | 71 | 0,732150324 |
| Thalamo_occipital_right | 72,785 | 65 | 0,78399392 |
| Thalamo_parietal_left | 181,364 | 97 | 0,86920906 |
| Thalamo_parietal_right | 171,652 | 102 | 0,855675489 |
| Thalamo_postcentral_left | 64,497 | 81 | 0,876537894 |
| Thalamo_postcentral_right | 57,052 | 87 | 0,874093112 |
| Thalamo_precentral_left | 84,726 | 83 | 0,85796197 |
| Thalamo_precentral_right | 81,604 | 86 | 0,876979252 |
| Thalamo_prefrontal_left | 154,055 | 68 | 0,876583081 |
| Thalamo_prefrontal_right | 155,472 | 64 | 0,799537217 |
| Thalamo_premotor_left | 60,046 | 63 | 0,789921589 |
| Thalamo_premotor_right | 52,226 | 61 | 0,728706312 |
| Uncinate_Left | 63,295 | 11 | 0,776357036 |
| Uncinate_Right | 47,888 | 9 | 0,846666671 |

*Supplementary Table 3. Regression coefficients from the full General Linear Model with CES-D score as the outcome variable, and clinical variables as predictor variables. * for p<0.05, ° for p<0.10.*

| **Predictor Variable** | **Estimate** | **SE** | **tStat** | **p-Value** |
| --- | --- | --- | --- | --- |
| (Intercept) | 20.906 | 3.738 | 5.593 | 6.29E-08* |
| **Female sex** | **1.382** | **0.697** | **1.983** | **0.049*** |
| Age | -0.021 | 0.021 | -0.978 | 0.329 |
| Living alone | 0.201 | 0.822 | 0.245 | 0.807 |
| Education level | -0.150 | 0.217 | -0.688 | 0.492 |
| NIHSS | -0.467 | 0.416 | -1.124 | 0.262 |
| mRankin | 0.906 | 0.558 | 1.624 | 0.106 |
| *MOCA* | *-0.199* | *0.117* | *-1.699* | *0.091°* |
| **Depression self-evaluation** | **2.002** | **0.368** | **5.446** | **1.32E-07*** |
| Anxiety self-evaluation | 0.015 | 0.151 | 0.101 | 0.919 |
| **EPICES** | **0.053** | **0.022** | **2.369** | **0.019*** |

*Supplementary Table 4. Regression coefficients from the full General Linear Model with CES-D score as the outcome variable, and radiological lobar locations as predictor variables. * for p<0.05, ° for p<0.10.*

| **Predictor Variable** | **Estimate** | **SE** | **tStat** | **p-Value** |
| --- | --- | --- | --- | --- |
| (Intercept) | 15.919 | 0.640 | 24.876 | 5.01E-67* |
| **Frontal** | **2.839** | **0.878** | **3.233** | **0.001*** |
| Parietal | -0.124 | 0.941 | -0.132 | 0.895 |
| Insular | 1.964 | 1.351 | 1.454 | 0.147 |
| Temporal | -1.888 | 1.226 | -1.539 | 0.125 |
| Occipital | 0.786 | 0.970 | 0.810 | 0.419 |
| Basal Ganglia | -0.332 | 0.827 | -0.401 | 0.689 |
| **Cerebellum** | **2.183** | **0.951** | **2.296** | **0.023*** |
| Brainstem | -0.577 | 1.173 | -0.492 | 0.623 |

*Supplementary Table 5. LASSO regression coefficients from the four non-zero atlas-based models: the gray-matter model, the white-matter model, the functional network model and the network topology model.*

| **ROI name** | **Beta estimate** |
| --- | --- |
| **Gray-matter regions model** | |
| ctx_lh_transversetemporal | 2,875 |
| ctx_rh_postcentral | 1,069 |
| ctx_rh_precentral | 0,476 |
| **White mater tract model** | |
| Handmid_U_tract_Left | 2,501 |
| **Functionnal networks model** | |
| SomatoMotor_dorsal | 1,450 |
| SomatoMotor_lateral | 1,251 |
| **Network topology model** | |
| max_betweenness_centrality | -3.946E-04 |
| SalVentAttn_efficiency | -22,022 |
| Cont_efficiency | -63,587 |
| Default_efficiency | -10,011 |

Supplementary Table 6. Hierarchical stepwise regression coefficients for the subordinate models. Predictions from subordinate models were normalized with Zscore such that regression coefficient can be interpreted directly as the weight of each subordinate model in the hierarchical prediction. ** for p<0.05.*

| **Model** | **Estimate** | **SE** | **tStat** | **p-Value** |
| --- | --- | --- | --- | --- |
| (Intercept) | 17.09 | 0.29 | 59.33 | 1.34E-140* |
| Clinical | 2.17 | 0.30 | 7.27 | 5.60E-12* |
| Radiological | 0.80 | 0.29 | 2.70 | 0.01* |
| Gray-matter | 0.71 | 0.30 | 2.37 | 0.02* |
| Network Topology | 1.04 | 0.29 | 3.60 | 3.91E-04* |


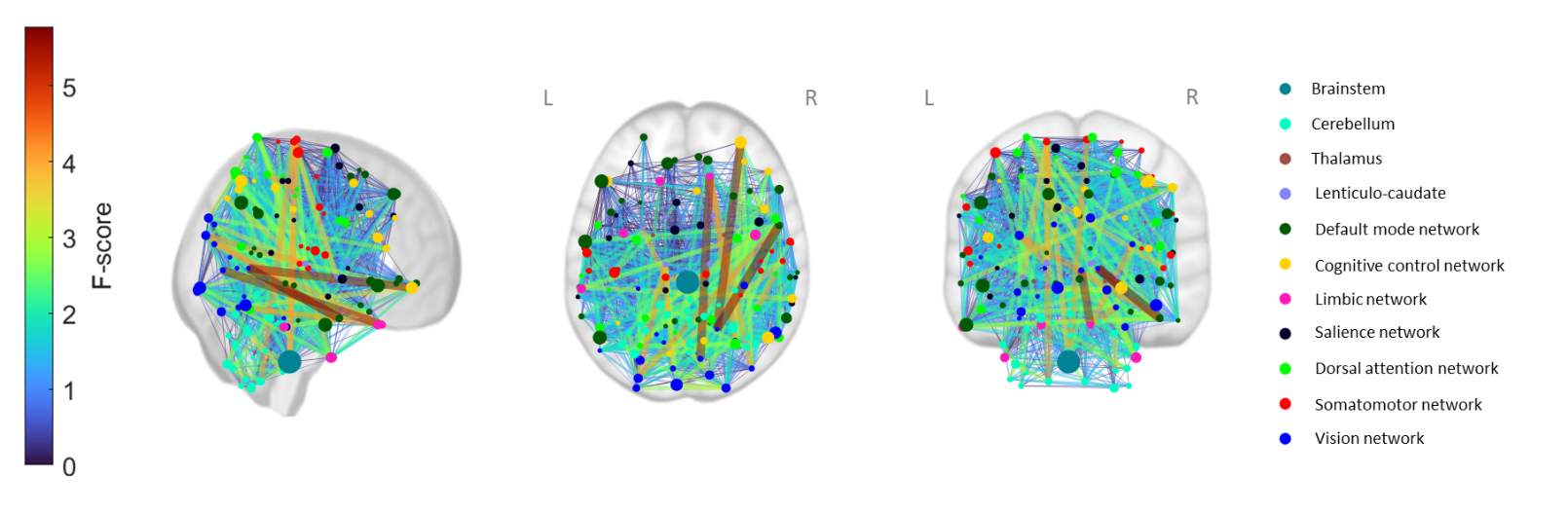


*Supplementary Figure 1. Unthresholded network-based-statistics depicting the whole-brain parcel-wise edges F-score.*


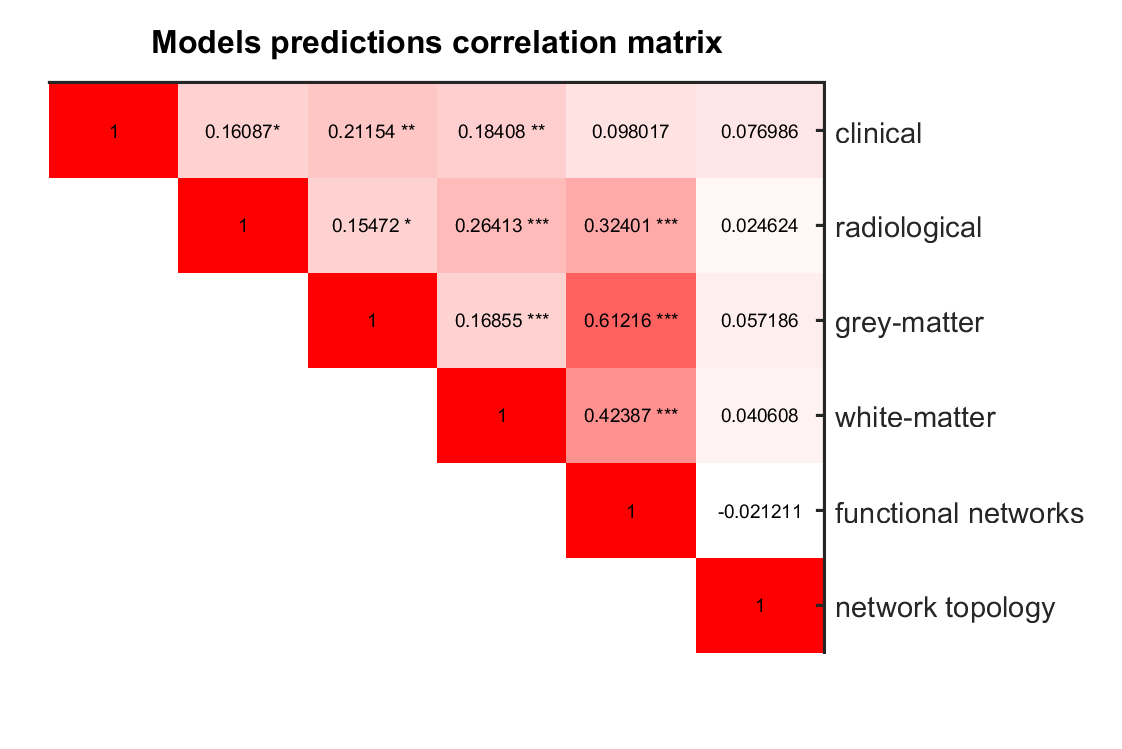


*Supplementary Figure 2. Models prediction correlation matrix, computed with pearson correlation coefficients. Signifance levels are depicted as followed: ° p<0.1, * p<0.05, ** p<0.01, *** p<0.001.*
